# Prediction Models for Evaluating Resilient Modulus of Stabilized Aggregate Bases in Wet and Dry Alternating Environments: ANN and GEP Approaches

**DOI:** 10.3390/ma15134386

**Published:** 2022-06-21

**Authors:** Kaffayatullah Khan, Fazal E. Jalal, Mohsin Ali Khan, Babatunde Abiodun Salami, Muhammad Nasir Amin, Anas Abdulalim Alabdullah, Qazi Samiullah, Abdullah Mohammad Abu Arab, Muhammad Iftikhar Faraz, Mudassir Iqbal

**Affiliations:** 1 Department of Civil and Environmental Engineering, College of Engineering, King Faisal University, Al-Ahsa 31982, Saudi Arabia; mgadir@kfu.edu.sa (M.N.A.); 218038024@student.kfu.edu.sa (A.A.A.); 219041496@student.kfu.edu.sa (A.M.A.A.); 2 Shanghai Key Laboratory for Digital Maintenance of Buildings and Infrastructure, State Key Laboratory of Ocean Engineering, School of Naval Architecture, Ocean & Civil Engineering, Shanghai Jiao Tong University, Shanghai 200240, China; jalal2412@sjtu.edu.cn (F.E.J.); or mudassiriqbal29@sjtu.edu.cn (M.I.); 3 Department of Structural Engineering, Military College of Engineering (MCE), National University of Science and Technology (NUST), Islamabad 44000, Pakistan; moak.pg18mce@student.nust.edu.pk; 4 Department of Civil Engineering, CECOS University of IT and Emerging Sciences, Peshawar 25000, Pakistan; 5 Interdisciplinary Research Center for Construction and Building Materials, Research Institute, King Fahd University of Petroleum and Minerals, Dhahran 31261, Saudi Arabia; salami@kfupm.edu.sa; 6 Department of Civil Engineering, University of Engineering and Technology, Peshawar 25120, Pakistan; samiullah.qazi@uetpeshawar.edu.pk; 7 Department of Mechanical Engineering, College of Engineering, King Faisal University, Al-Ahsa 31982, Saudi Arabia; mfaraz@kfu.edu.sa

**Keywords:** AI modelling, resilient modulus, pavements, wet–dry cycles, sensitivity analysis, parametric study

## Abstract

Stabilized aggregate bases are vital for the long-term service life of pavements. Their stiffness is comparatively higher; therefore, the inclusion of stabilized materials in the construction of bases prevents the cracking of the asphalt layer. The effect of wet–dry cycles (WDCs) on the resilient modulus (M_r_) of subgrade materials stabilized with CaO and cementitious materials, modelled using artificial neural network (ANN) and gene expression programming (GEP) has been studied here. For this purpose, a number of wet–dry cycles (WDC), calcium oxide to SAF (silica, alumina, and ferric oxide compounds in the cementitious materials) ratio (CSAFRs), ratio of maximum dry density to the optimum moisture content (DMR), confining pressure (σ_3_), and deviator stress (σ_4_) were considered input variables, and M_r_ was treated as the target variable. Different ANN and GEP prediction models were developed, validated, and tested using 30% of the experimental data. Additionally, they were evaluated using statistical indices, such as the slope of the regression line between experimental and predicted results and the relative error analysis. The slope of the regression line for the ANN and GEP models was observed as (0.96, 0.99, and 0.94) and (0.72, 0.72, and 0.76) for the training, validation, and test data, respectively. The parametric analysis of the ANN and GEP models showed that M_r_ increased with the DMR, σ_3_, and σ_4_. An increase in the number of WDCs reduced the M_r_ value. The sensitivity analysis showed the sequences of importance as: DMR > CSAFR > WDC > σ_4_ > σ_3_, (ANN model) and DMR > WDC > CSAFR > σ_4_ > σ_3_ (GEP model). Both the ANN and GEP models reflected close agreement between experimental and predicted results; however, the ANN model depicted superior accuracy in predicting the M_r_ value.

## 1. Introduction

The concept of durability is interwoven with the functionality of stabilized paving materials [1]. Aggregates, water, cementitious ingredients, and/or emulsified asphalt make up stabilized base or subbase components [2,3]. A decent riding surface and waterproofing mechanism in case of the base course are provided by an asphalt-wearing course material [4,5], and the quality of material as well as the thickness of granular layers determine the life period of a thin asphalt pavement. Because most building materials have a finite lifespan owing to wear and tear, more creative, inventive, cost-effective, and environmentally friendly highway design solutions are required [6]. As a result, there is a pressing need to reduce the cost of constructing and preserving the national transportation infrastructure [7]. Due to their comparatively greater stiffness in contrast to a variety of conventional materials, the incorporation of stabilized materials in the construction of bases tends to prevent failure-related cracking inside the asphalt layer [8]. According to Kaloop et al. [3], these reflective cracks in the asphalt layer are frequently caused by the origination of cracking in the stabilized base layer. Note that a stabilized base layer with correct design and construction would persist through asphalt maintenance and/or overlays, and this layer may be utilized instead of a conventional base layer or below a conventional base layer. Stabilized materials must be sufficiently stronger and longer-lasting to withstand traffic and climatic variations, particularly wet–dry cycles (WDCs), as well as the freeze–thaw cycles (FTCs) [5]. As per the mechanistic empirical pavement design guidelines (MEPDG) and the Swedish design model ERAPAVE, the WDCs and FTCs are considered vital in the degradation of the base/subbase materials, resulting in premature pavements failure, among various parameters [9,10,11]. In the case of seasonally frozen soils, the FTCs are categorized by an interrupted temperature variation that has a significant impact on geotechnical engineering. The impact of FTCs on soil is governed by the amount of moisture. Larger moisture contents tend to highly deteriorate the soil structure because of the phase change of water [12]. After 4 weeks of curing the samples, 12–30 WDCs may be adequate, and a number of cycles exceeding 30 is essential for 3-day-cured samples. Furthermore, the beneficial effect of curing duration was stronger on 3-day-cured specimens, while the negative impact of the WDCs was more intense in the case of 4-week-cured samples [13]. Avirneni et al. [14] proved that the detrimental effects of wet dry cycles ceases incorporating Reclaimed Asphalt Pavement and Fly Ash in base course. According to Sobhan and Reddy [15], the specimens exposed to WDCs incurred comparatively much more damage, as measured by permanent deformation, residual compressive, and ultimate strength values, as well as their resistance to wear. Furthermore, a link between WDC strength and unsoaked strength was proposed by Kampala et al. [16], since the durability is observed to be in close association with the unsoaked strength prior to WDCs.

According to Kaloop et al. [3], there exists substantial number of relationships among WDCs and FTCs from the standpoint of durability as well as the resilient modulus (M_r_). The M_r_ determines the efficacy of base materials in various pavement structures [17,18]. The M_r_ values of 4-week-cured samples treated to 30 cycles were approximately 5% less than the corresponding M_r_ values of samples experiencing no WDCs [5]. The M_r_ helps to model the subgrade behavior and is generally computed in the laboratory, as per the AASHTO T307 standard [19], or can be estimated using artificial intelligence (AI) techniques [20,21,22]. Furthermore, the M_r_ can be experimentally determined using the cyclic triaxial test results, which are defined by the ratio of deviator stress to resilient strain after load cycles [23]. Several experiments have been performed by past researchers to assess the impact of the WDCs on the M_r_ of stabilized base materials [3,24,25,26]. It was revealed that blending with certain additives (exhibiting cementitious nature) enhanced the long-term mechanical characteristics of the treated samples extracted from base materials in cases with WDCs, thereby increasing the modified M_r_ value [3]. However, Khoury and Zaman [5] suggested a regression model to estimate the M_r_ of stabilized base aggregates on the basis number of WDCs, ratio of oxide ingredients in the cementitious materials, the physical characteristics of the mixture, and the various stress levels. Maalouf et al. [27] deployed support vector regression (SVR) for modelling the M_r_ of stabilized base aggregates exposed to WDCs, and it was concluded that the SVR approach outclassed both the regression and the least square techniques. Pourtahmasb et al. [28] performed an M_r_ prediction of asphalt mixtures comprising recycled concrete aggregate with the help of an adaptive neuro-fuzzy approach. It was revealed that the highest predictive performance and fitness of generalization was attained in cases of stone mastic asphalt, which comprised recycled concrete aggregates. Oskooei et al. [29] studied the incorporation of MLP in the form of substructure of an artificial neural network (ANN) technique by considering a detailed database obtained from the available literature to forecast the M_r_ of recycled aggregates. The proposed ANN models are thought to be cost-effective methods for reducing the experimental testing; however, one of the primary drawbacks of utilizing ANN for prediction is that it operates in a black box and does not produce a formula that can be used in the future. Gabr et al. [30] incorporated a novel technique to predict the M_r_ by incorporating extreme learning machine equilibrium optimizer methods. The results show that the extreme learning machine (ELM) and equilibrium optimizer (EO) (ELM-EO) and ELM–biogeography-based optimization (BBO) (ELM-BBO) techniques outperformed the ELM–genetic algorithm (ELM-GA) and regression approaches in terms of predicting the M_r_ value. Kayadelen et al. [23] performed numerical simulation as well as a novel methodology to compute the M_r_ in cases of traffic loading on a pavement embankment. In terms of training performances and prediction accuracies, statistical performance assessments revealed that the random forest (RF) model greatly surpassed the M5P models. The numerical study revealed that mechanical characteristics such as elastic modulus are the most important factors influencing the behavior of materials subjected to repetitive loads. Kezhen et al. [31] estimated the M_r_ of an asphalt pavement material with the help of SVM. The results show that the proposed SVM model can predict M_r_ and other mechanical behavior indexes of asphalt pavement material with greater precision in comparison with the ANN method and multiple regression.

For the modelling of engineering applications, extremely powerful learning algorithms have recently been created. The currently formulated AI approaches include the ANNs (subtypes: Bayesian neural network [32], general regression neural network [33], back-propagation neural network [34], k-nearest neighbor [35], multilayer perceptron neural network [36]) and the hybrid forms of ANNs (i.e., adaptive neuro-fuzzy inference system (ANFIS) [22,37,38,39,40]). In addition, the ANN, the particle swarm optimization algorithm (PSO), and gene expression programming (GEP) are extremely beneficial techniques used to formulate a variety of prediction models. The ANN has been extensively utilized for the estimation of the M_r_ values in cases of pavement materials [41,42,43,44,45,46,47]. The ANNs are AI-inspired biological neural networks and problem-solving machine learning models that mimic the cellular structures of the human brain and nervous system. They directly take into consideration the relationship between the model input variables and the corresponding outputs without giving simple mathematical expression, thus inhibiting their practical implications; however, their degree of accuracy is comparatively higher [48,49,50,51,52,53,54,55,56]. On the other hand, Cramer invented genetic programming (GP) in 1985, which was ameliorated with the help of various shapes and sizes. Additionally, the GEP was invented by C. Ferreira twenty years ago. It comprises simple, linear chromosomes with fixed lengths, which encode a program and exhibit the capability to estimate cumbersome and highly nonlinear problems in order to evaluate regressions, modelling functions, forecasting, and detecting in data mining. The GEP models are successful as they yield easy-to-use convenient mathematical formulae to compute the output value [22,57,58,59,60,61,62,63]. ANN, ANFIS, and GEP techniques were deployed to determine the swell pressure and the unconfined compression strength of swelling to compare the accuracy of the aforementioned AI methods and their performances, with special focus on the GEP method. The overall coefficient of correlation values followed the order ANN > GEP > ANFIS, such that all the R-values exceeded 0.80. In addition, the GEP model outclassed the ANN and ANFIS techniques in terms of the closeness of the training, validation, and the testing datasets [22]. Undertaking resilient modulus testing is expensive, time-consuming, and complex. The M_r_ of compacted subgrade soils was predicted under influences of freeze–thaw cycles and moisture using the GEP and ANN approaches. The formulated GEP and ANN models computed the M_r_ value and attained superior performance in comparison with a variety of other empirical models [29,64]. While determining the elastic modulus of soil, the accuracy of the developed ANN model was superior (R^2^ of 0.98) and it supersedes the multiple regression model developed using the same data. The performance comparison revealed that the ANN model could be used to estimate the modulus of elasticity of soil with more confidence [65]. In order to examine the efficacious stabilization of extremely weak subgrade soils at high water contents, the resilient modulus of stabilized subgrade was determined; therefore, ANN and GEP models were formulated by considering 125 samples data and it was concluded that accurate result for M_r_ was achieved by using GEP (R^2^ of 0.95) [17]. In yet another study regarding prediction of M_r_, the computation of a rolling-wheel deflectometer and a falling weight deflectometer was yielded from a testing program for training an ANN-based model, which was independently validated using data from a testing program, such that it depicted an acceptable accuracy in both the development and validation phases (R^2^ of 0.73 and 0.72, respectively) [66]. Moreover, Jalal et al. [57] suggested that the genetic programming approaches (i.e., GEP and MEP) techniques accurately forecast the compaction characteristics (maximum dry density and optimum moisture content) of swelling clays, such that the GEP model showed a relatively better performance.

Despite the fact that ANN and GEP have been shown to be effective approaches for modelling a variety of engineering applications, there has been little research on modelling M_r_ in pavement applications. There is a dire need for pavement engineers and practitioners to deploy easy-to-use mathematical expressions for the design phase or on site without the need to conduct laborious and expensive laboratory testing. Therefore, in order to discover a near-global solution for improved network prediction and to maintain high generalization capabilities of the network, ANN and gene expression programming (GEP) were deployed in the current study to assess the M_r_ of pavement materials. The central aim of this research study was to formulate and design ANN and GEP models for predicting the M_r_ of stabilized base aggregates subjected to WDCs on the basis of the M_r_ data presented. The robustness of the ANN and GEP models was statistically evaluated and validated to forecast the M_r_ of stabilized base aggregates. The structure of this article in the following sections comprises a collection of the experimental database; overviews of the ANN and GEP algorithms; the modelling of Mr using these AI techniques; sensitivity and parametric studies; performance evaluation of the developed ANN and GEP models; and the conclusion.

## 2. Research Methodology

### 2.1. Experimental Database

**Figure 1:**
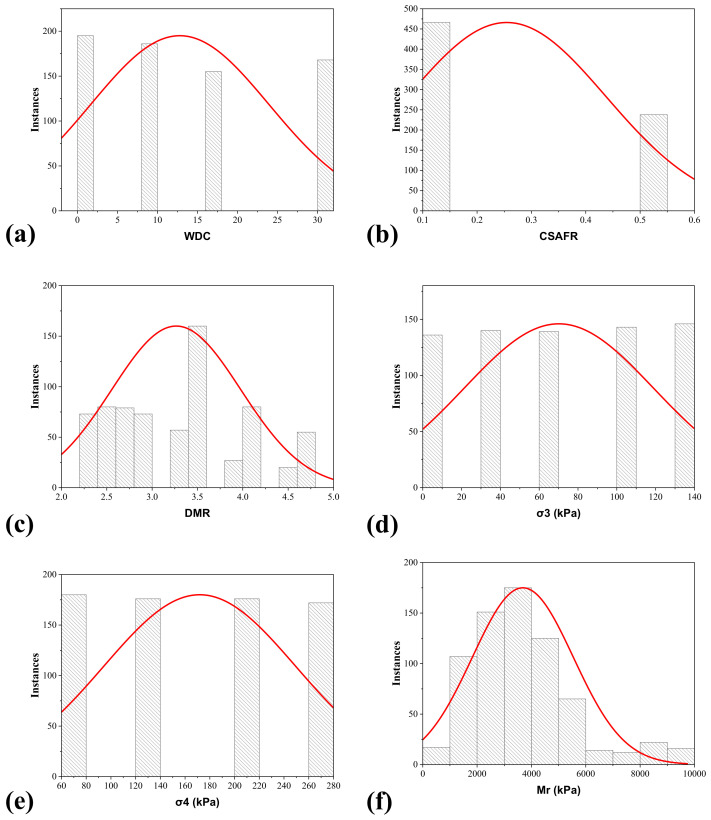
Distribution histogram of the variables considered in the current study: (**a**) number of wet–dry cycles (WDC), (**b**) calcium oxide to SAF (silica, alumina, and ferric oxide compounds in the cementitious materials) ratio (CSAFR), (**c**) ratio of maximum dry density to the optimum moisture content (DMR), (**d**) confining pressure (σ_3_), (**e**) deviator stress (σ_4_), and (**f**) target parameter, i.e., resilient modulus (M_r_).

For developing strong and robust AI models, it is crucial to generate a well-assembled and extensive dataset, whose description is clear and precise, with clear insights, and where the considered input variables are statistically significant. Therefore, a brief database containing records of 704 stabilized aggregate bases experimental tests [3] was utilized to train the two algorithms (i.e., ANN and GEP) chosen for this investigation. The dataset consists of input parameters (i.e., number of wet–dry cycles (WDC), calcium oxide to SAF (silica, alumina, and ferric oxide compounds in the cementitious materials) ratio (CSAFR), ratio of maximum dry density to the optimum moisture content (DMR), confining pressure (σ_3_), and deviator stress (σ_4_)) and target parameters (i.e., resilient modulus (M_r_)). The input and the target parameters and their individual data have been described in Table 1. In addition, Figure 1 shows the histogram distribution plots of the input variables and target parameters used during the ANN and GEP models training. Plotting these values may help to identify parameters exhibiting inadequate data; therefore, additional data are required (Asteris et al., 2021). All the analyzed input variables and target variables were correlated using the Pearson correlation coefficient (r), and the results are shown in Table 2. A brief examination of the dependence between the input variables and the target variable revealed that all the inputs are positively correlated except the WDC, which is negatively correlated (r = −0.29605). CSAFR and the DMR showed strong positive correlation (i.e., 0.457157 and 0.714551, respectively), σ_3_ and σ_4_ (0.076791 and 0.137871, respectively) showed moderate positive correlation, and WDR showed a moderate negative correlation (r = −0.29605) with the M_r_ of the stabilized aggregate bases.

### 2.2. Overview of ANN

Artificial neural networks (ANNs) are straightforward yet reliable computational models. They attempt to mimic the human nervous system and brain to solve a given task. In recent times, ANNs have increasingly been used for numerous engineering applications [22,67,68]. ANN-based algorithms have also been successfully implemented for different geotechnical engineering problems, such as soil stabilization [69], slope stability analysis [70], and foundation settlement predictions [71]. A comprehensive explanation of ANNs is beyond the scope of this study. Many previous studies have described the structure and functioning of ANNs [72,73]. A typical ANN structure comprises several processing elements (also called nodes) that are often organized in different layers, for instance; an input layer, an output layer, and one or more hidden layers. Multilayer networks are more robust than single-layer networks. The optimum hidden layer in ANNs may be determined using the trial-and-error technique. The input from the previous layer $\left(x_{i}\right)$ from each node is multiplied by a modifiable connection weight $\left(w_{ji}\right).$ At each node, weighted input signals are added. A threshold value $\left(\phi_{j}\right)$ is also added at this stage. A nonlinear transfer function $\left(f(.\right))$ is then applied to this joint input $\left(I_{j}\right)$ to generate the node output $\left(y_{j}\right).$ The transfer functions normally used are linear, sigmoidal, and/or their combination. The output of one layer serves as input for the nodes in the next layer, and the process is iteratively repeated. The operation of ANNs is summarized in Figure 1, and the following relations (Equations (1) and (2)) present the aforementioned process:

(1) $$I_{j}=\overset{n}{\underset{i=1}{\sum }}w_{ji}+\phi_{j}$$

(2) $$y_{j}=f\left(I_{j}\right)$$

Information propagation in ANNs commences at the input layer where data are fed. The system weights are then adjusted iteratively using learning rules to find the optimal set of weights. The procedure for adjusting the connection weights is known as “training”. It is pertinent to mention that the Levenberg–Marquardt backpropagation is the most frequently used training method for multilayer networks. The stopping criterion is an important aspect of the ANNs model, which determines whether the model has been trained sufficiently. Model training is stopped based on two criteria: (i) if there are slight changes in the training error with increasing iterations; and/or (ii) if it reaches a sufficiently small value. However, studies have reported that adopting such techniques for stopping criteria may lead to overtraining issues or premature stopping. To overcome this problem, application of the cross-validation method has been proposed that involves splitting the data into three distinct sets, i.e., training, testing, and validation. ANNs use the training set (biggest among these) to identify patterns in the data. Network training aims to determine the set of weights $w_{ji}$ between the neurons to obtain the global minimum of the error function according to the following relation (Equation (3)). The primary purpose of the testing set is to assess the generalization capability of the trained network while its final check is conducted using the validation dataset.

(3) $$y_{j}^{k}=f\left(\overset{nk-1}{\underset{j=1}{\sum }}w_{ji}^{k}+y_{j}^{n-1}\right)$$

For the current study, ANNs were preferred due to their apparent advantages over other data mining techniques. The application of ANN is advantageous from various fronts, such as effective and efficient data analysis, their superior abilities to handle both complex and nonlinear problems, and their reliable predictions.

### 2.3. Overview of GEP

**Figure 2:**
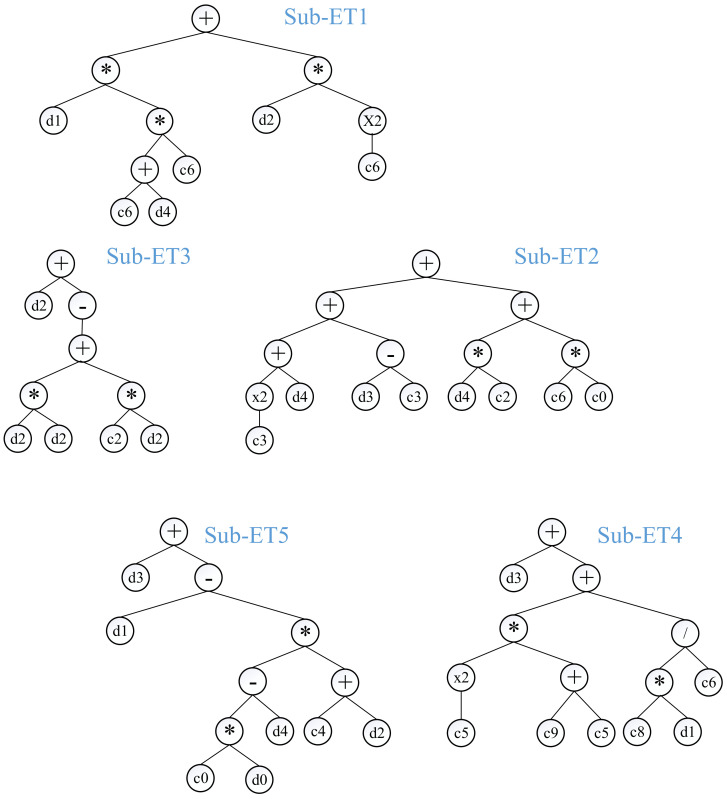
Expression trees (ETs) of the GEP model in the current study (* denotes multiply sign, / denotes division, +, and − denote addition and subtraction, respectively).

Gene expression programming (GEP) was initially proposed by Koza (1992), and is inspired by Darwin’s theory of evolution and natural selection. GEP has been successfully implemented for solving various geotechnical and geological applications. Like traditional GAs and GPs, GEP uses a population-based strategy to solve prediction problems. The process is initialized with random generation of individuals, followed by induction of genetic variations in the parent population using genetic operators (crossover, elitism, mutation), finally selecting the offspring based on their fitness values. Figure 2 presents the main steps involved during the prediction through the GEP. All the steps are successively applied to diversify and enrich the offspring population. The fundamental difference between the two algorithms is the nature of individuals/populations. In GA, individuals represent the linear strings with fixed lengths (chromosomes); in the GP, these imply nonlinear entities of different shapes and sizes (parse trees); in the GEP, the individuals are first encoded as fixed-length linear strings (genome), which are subsequently expressed as nonlinear entities of various shapes and sizes (expression trees or ETs). This hybridization strategy of GEPs makes them extremely versatile and reliable compared with other existing evolutionary methods. A GEP model is built on input variables, arithmetic operations, and mathematical formulations. The predictive performance of a GEP algorithm is highly dependent on model parameters, such as the number of chromosomes (candidates models), the number of genes (indicating subsections of candidate models), linking function (used for connecting subsections), head size (shows the complexity of a subsection), mutation and crossover (genetic operators), and the maximum number of generations. Termination conditions are employed to evaluate whether the model performance has achieved the expectations of the predictions. The head in the GEP model comprises symbols for representing both functions and terminals. For a given prediction problem, the length of tail “*t*” is calculated as a function of the length of head “*h*” using a maximum number of arguments (*n*) of the function according to the following expression Equation (4):

(4) $$t=h\left(n-1\right)+1f$$

The GEP model was selected for the analysis because the method can provide a simple mathematical prediction model that may be used by practitioners in the field with high confidence for other similar problems.

### 2.4. AI Modelling

Two AI approaches, i.e., ANN and GEP, were used to estimate the resilient modulus (M_r_) of stabilized aggregate bases, as previously mentioned. Because of its high accuracy and quick convergence characteristics, the Levenberg–Marquardt backpropagation technique was employed to train the ANN model with 70% of the data [74]. Several experimental trials were conducted with one, two, and three hidden layers and multiple neurons. As shown in Table 3, the best results were obtained employing a single hidden layer with 10 neurons. Between training and validation, the data was split up on a random basis. The anticipated yield was assessed using the correlation coefficient R. A single hidden layer of neurons was utilized to predict the resilient modulus using five inputs supplied in the form of five neurons.

Using a defined number of input variables, the GEP algorithm generates a basic mathematical model to estimate a single target variable [61]. Moreover, the purpose of this study was to formulate a mathematical model that could account for the M_r_ of stabilized aggregate bases using the input features. Additionally, in the case of training the GEP model, the data were split into training and validation subsets. For the changeable setting factors, such as the number of genes, chromosomes, and head size, the trial-and-access approach was employed to achieve the hyperparameters of the GEP model [75,76,77]. Using the MAE, RSE, and RMSE as fitness functions, the setup parameters were altered according to Table 4. After training, the performance of the developed GEP model was evaluated. Additionally, the GEP models were evaluated using two main indices, i.e., R and MAE. Table 4 shows the five models that were generated in the current study. With the R value considerably more than 0.8 for all the training and validation datasets, all the proposed GEP models were in close agreement with the actual datasets. The model constructed utilizing 30 chromosomes with 8 head sizes and 5 genes, on the other hand, had the strongest correlation among the lowest R and MAE values in both the training and validation stages. As a result, the GEP model attained in the third trial (Table 4) was utilized to generate a mathematical equation based on the ETs (Figure 2) and the MATLAB model obtained from the modeling process. To generate the ETs, function sets were used where addition was selected to link these trees. It was also discovered that increasing the complexity of the function set boosted the model’s resilience; however, this increased the complexity of the output equation. As a result, the model was given a basic function set.

## 3. Results and Discussions

### 3.1. Comparison between Predicted and Experimental Results

**Figure 3:**
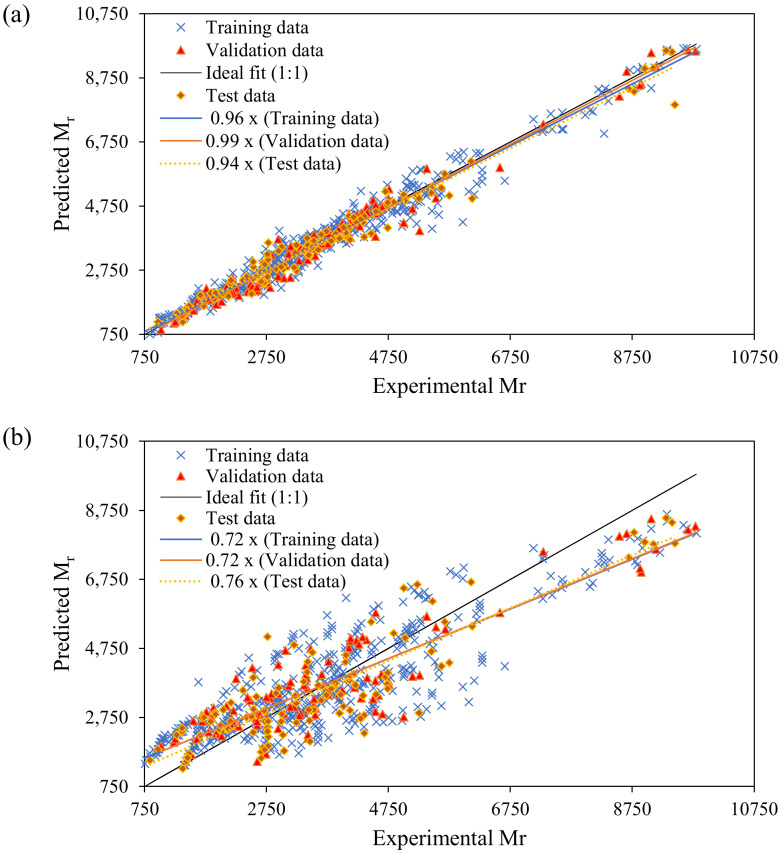
Comparison of experimental and predicted results: (**a**) ANN; (**b**) GEP.

This subsection deals with the comprehensive analysis of the proposed models using ANN and GEP for the prediction of M_r_, based on the slope of the regression line for all the three datasets, i.e., training, validation, and testing set. The performance of the proposed models depends on the closeness of the datapoints to the regression line [78]. For a good model performance, the slope of the regression line must be nearer to unity, and equal for an ideal fit. It can be clearly seen in Figure 3a that, for ANN, the slope of regression line of the training, validation, and testing sets are 0.96, 0.99, and 0.94, respectively, which are nearer to an ideal fit (1:1). However, for GEP model, these values are 0.72, 0.72, and 0.76, respectively (Figure 3b). As shown in the figures, the variation of experimental and predicted output by ANN is close to the 45° line. The ANN gives an outburst performance with slope of best-fitted line, nearly equaling unity. However, in the testing stage, it can be recorded that the slope is considerably reduced. The GEP is better in terms of the closeness of the slope in all the three stages. The dispersion of the M_r_ shows that both the proposed models accurately consider the influence of all five input variables to predict M_r_, which possesses a strong correlation and lower biasness [59,79]. The variation of the datapoints over its range further shows that there is no overfitting issue in all the developed models [80,81].

**Figure 4:**
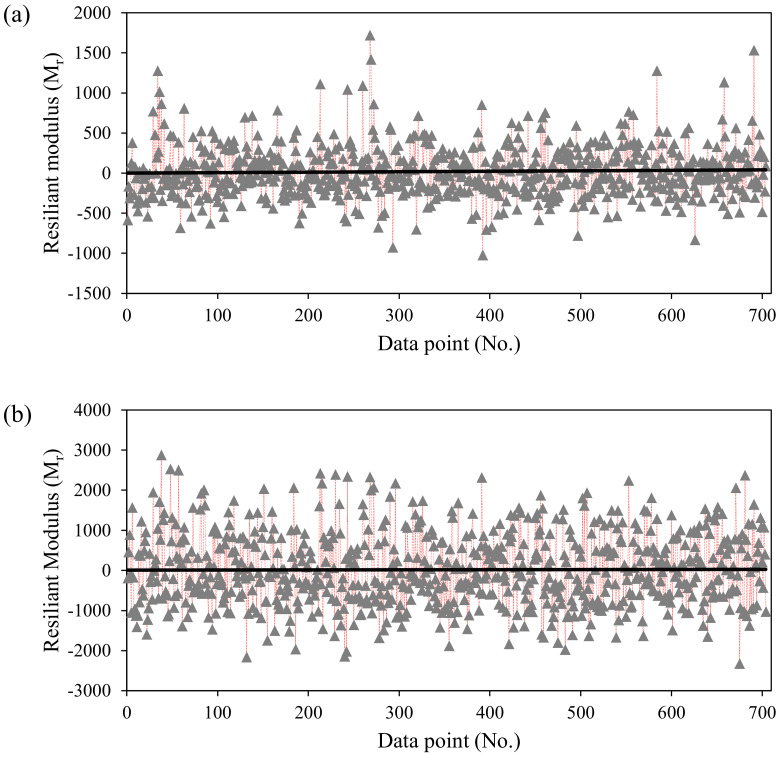
Error analysis of the proposed models: (**a**) ANN; (**b**) GEP.

Furthermore, the error values of each individual data point used for the prediction of M_r_ using ANN and GEP are graphically presented in Figure 4. The maximum positive and maximum negative errors recorded are 2879.68 and −2323.25, respectively (in the case of ANN), and 1722.21 and −1020.7, respectively (in the case of GEP). For a given wide range of experimental records, a total of 98% and 78% datapoints have an error value in range [–1000, 1000] for ANN and GEP models, respectively. It shows that the error values of the ANN model are mainly scattered around zero, which depicts its robust performance. Like the slope of the regression line, error analysis also shows the comparatively better performance of the ANN model followed by the GEP approach [22].

### 3.2. Formulation of M_r_

Seven GEP models were derived with varying fitness functions (i.e., RMSE, MAE, and RSE), number of chromosomes, genes, and head size. The best-performing model based on R and MAE with 30 chromosomes, 5 genes, and 8 head size was retrieved for further validation. In Figure 2, the best-performing GEP model is shown in the form of ETs in order to deduce an empirical equation for determining the M_r_. As illustrated, sub-ETs (1–5) have four basic mathematical functions: +, −, ÷, and ×. After decoding the sub-ETs, the GEP equation obtained is explicitly presented in Equations (5)–(10), which could be used for estimating the M_r_. Based on the number of datapoints, the developed model satisfies the minimum required limit for an ideal model, and is reliable and effective for estimation of M_r_ [82,83,84].

(5) Mr=A+B+C+D+E

(6) $$A=\left(\mathrm{CSAFR}\times \left(129.73+\left(11.39\times \sigma_{4}\right)\right)\right)+\left(129.73\times \mathrm{DMR}\right)$$

(7) $$B=85.86+\sigma_{4}+\sigma_{3}-\left(6.54\times \sigma_{4}\right)$$

(8) $$C=\;\mathrm{DMR}\times \left({\left({\mathrm{DMR}}^{2}+\left(1.74\times \mathrm{DMR}\right)\right)}^{2}+2.78\right)$$

(9) $$D={\;\sigma }_{3}+1592.14+\left(46.45\times \mathrm{CSAFR}\right)$$

(10) $$E={\;\sigma }_{3}+\mathrm{CSAFR}-\left(\left(\left(6.78\times \mathrm{WDC}\right)-\sigma_{4}\right)\times \left(3.18+\mathrm{DMR}\right)\right)$$

### 3.3. Importance of Input Variables

This section deals with the ultimate effect of selected input variables on the M_r_ based on ANN and GEP established models. The prime objective of conducting a sensitivity study is to investigate how the uncertainty regarding an outcome of the mathematical models or systems can be assigned to different uncertain sources [57,85]. Mostly the efforts in the wide area of ANN studies have concentrated on leading to formation of additional rules and procedures for training, enhancing network design, and expanding into unique domains of ANN applicability. However, there has been insufficient research on the acquisition of deep information for understanding the structure of back-end processing and the inner interpretations produced during ANN modelling in response to a particular complex problem. The ANNs are often portrayed to their users and clients in the form of black boxes with complex internal architectures that work to transform inputs into desired outputs. For neural networks of significant complexity levels, it is not generally feasible to determine or comprehend the precise processes behind the activation levels of hidden neurons or the weights of an ANN network in relation to the issue under investigation. Thus, determining the association between every explanatory variable and every response parameter in an ANN has always been a challenging task [86]. In the current research, relative importance (ranking) analysis for input variables utilized in the ANN modelling was performed based on significance of weights utilizing the technique provided in past studies [86,87], which can also be shown in Equation (11). The training dataset was used for the sensitivity analysis and the significance of the weights. In addition, the Milne’s approach was solely used to the connection weights in the ANN network.

(11) $$IIF\left(\%\right)=\frac{\sum_{i=1}^{hid}\frac{W_{ij}}{\sum_{j=1}^{in}\left|W_{ij}\right|}\times W_{oi}}{\sum_{k=1}^{out}\left(\sum_{i=1}^{hid}\left|\frac{W_{ik}}{\sum_{i=1}^{in}\left|W_{ij}\right|}\times W_{oi}\right|\right)}$$

In the above equation, $IIF\left(\%\right)$ is the importance of input variables in percentage; “in“, “out“, and “hid“ denote the number of inputs, outputs, and hidden layers, respectively. The process of recalculating the output, while considering substitutional assumptions, to find the impact of inputs using the sensitivity study is efficacious in ANN modelling for determining the back-end relation between inputs and between response parameters in a developed model [87].

Unlike the ANN, the GEP algorithmic structure provides a simple mathematical equation that helps in conducting the sensitivity of the proposed model using Equations (12) and (13) to judge the influence of input variables on the M_r_ value.

(12) $$R_{i}=f_{max}\left(y_{i}\right)-f_{min}\left(y_{i}\right)$$

(13) $$IIF\left(\%\right)=\left(\frac{R_{i}}{\sum_{n}^{j=1}R_{j}}\right)\times 100$$

where $f_{max}\left(y_{i}\right)$ and $f_{min}\left(y_{i}\right)$ are the maximum and minimum predicted M_r_ values for the $i^{\mathrm{th}}$ input domain. While calculating the $R_{i}$, all other inputs were maintained equal to unity.

**Figure 5:**
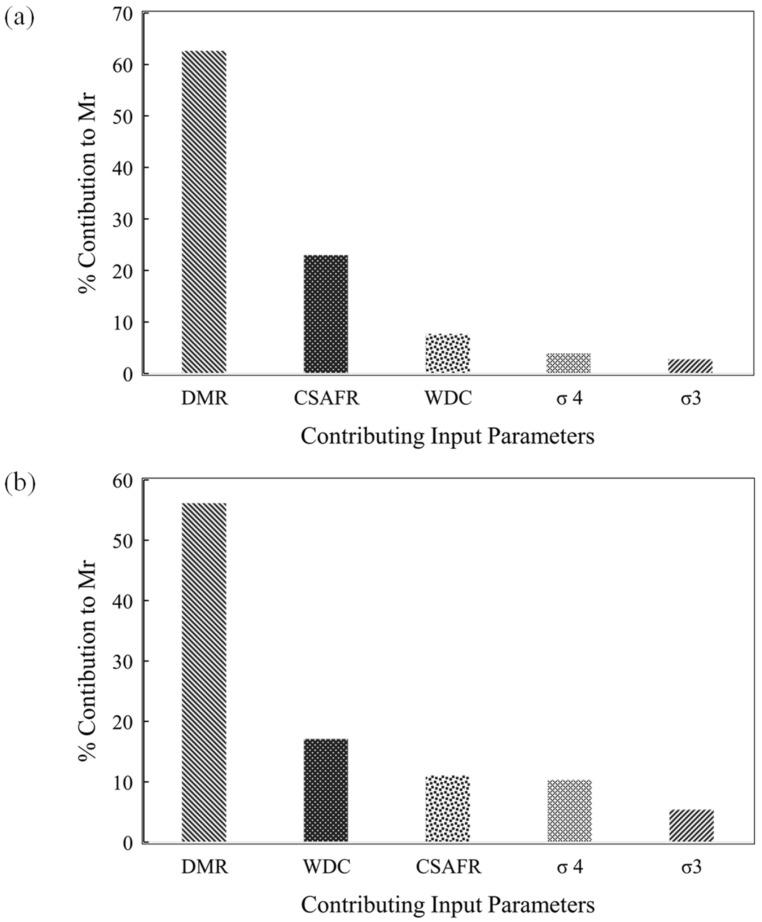
Importance of the variables reflected from (**a**) the ANN model and (**b**) the GEP model.

Figure 5a,b represent the relative importance of each input on the M_r_ which is reflected from the developed ANN and GEP models. Note that DMR is the most influential input in both the ANN and the GEP models. The increasing trend of inputs considering their influence on the M_r_ in the ANN model is DMR (62.63%) > CSAFR (22.96%) > WDC (7.72%) > σ_4_ (3.89%) > σ_3_ (2.77%). However, for the GEP model the importance of input variable follows the trend: DMR (56.13%) > WDC (17.08%) > CSAFR (11.05%) > σ_4_ (10.33%) > σ_3_ (5.39%).

### 3.4. Parametric Study

**Figure 6:**
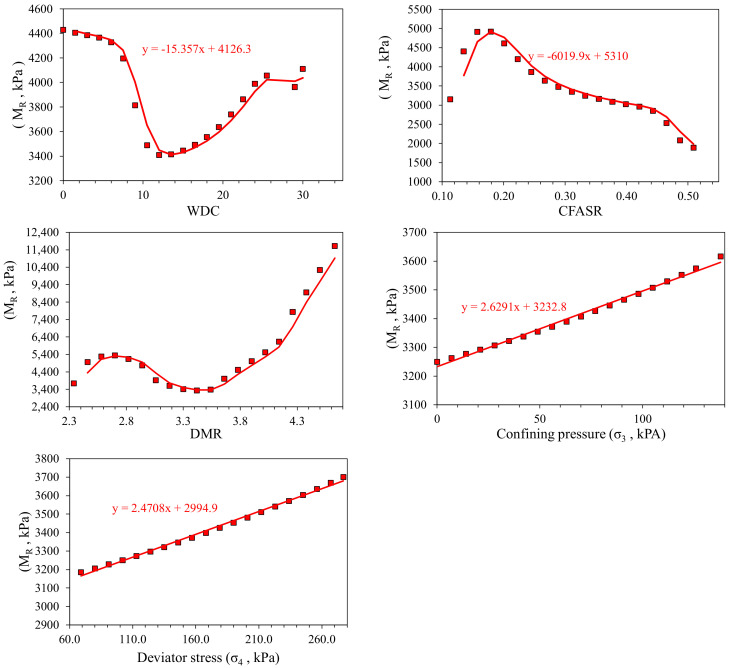
Parametric study of the ANN model.

**Figure 7:**
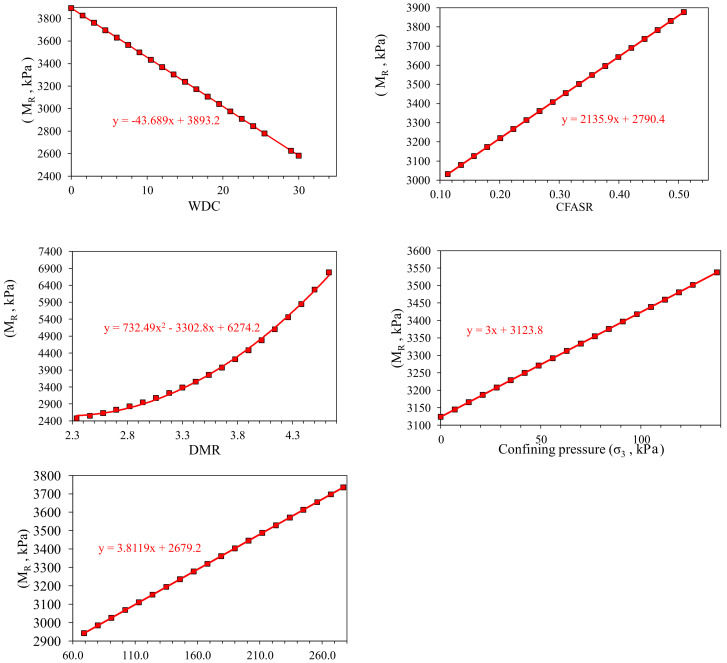
Parametric study of the GEP model.

It is important to check and verify the robustness of the developed ANN and GEP models using parametric analysis. The trends of the response parameter (M_r_) were assessed against the input variables and verified using the experimental results in the dataset to obtain the models with higher degree of accuracy and competence level [88,89]. Figure 6 (ANN) and Figure 7 (GEP) portray the expected increase in the M_r_ with an increase in DMR, σ_3_, and σ_4_. On the other hand, in both the models, the predicted M_r_ decreases with WDC. In addition, in the case of ANN, an increased CFASR resulted in a decreasing trend of M_r_. Conversely, in the GEP, the reverse trend is observed, i.e., increased CSFAR resulted in an increased M_r_. The simulated variations in the M_r_ with changes in the inputs are consistent and in line with trends in the actual experimental data, indicating the robustness and accurateness of the established ANN and GEP models.

### 3.5. Performance Evaluation of the Models

It is important to mention that the models attaining higher accuracy in the validation stage are more accurate, reliable, and robust [90,91]. The higher predictive accuracy in the training stage does not ensure the better performance of the models [57,92]. The performance of the AI models also depends on the number of data points and total number of inputs (independent variables) used for the prediction output (dependent parameter). The minimum acceptable ratio between the number of experimental records (data points) and the independent variables is 3 and preferably higher than 5, as suggested by Frank et al. [93,94]. In the current research, the ratios for the training, testing, and validation subsets were equal to 492/5 = 98, and 106/5 = 21 each, respectively, which were considerably greater than the prescribed limit in the literature.

The slope of the regression line for both model gives a broader knowledge of the variation of the data points around the 45° line [95,96]. As explained in Section 3.1 (Figure 3), neither of the developed models have any overfitting issues. Furthermore, the literature proposed that R depict the linear reliance of output and input variables, and must be greater than 0.8 for a strong correlation between experimental and predicted results [90,97]. However, R is insensitive to multiplication and division and cannot be used solely to assess the overall functioning of the models [22,98]. Thus, to assess the performance of the developed ANN and GEP model for the prediction of M_r_, the detailed statistical analysis using the correlation coefficient (R), the mean absolute error (MAE), the root squared error (RSE), and the root mean squared error (RMSE) is provided in Table 5 for the three subsets of each model.

#### 3.5.1. ANN Model

The ANN model is efficient in solving complex nonlinear engineering problems and provides higher accuracy. As presented in Table 5, the R_ANN_ nearly equals unity in training (0.983), testing (0.986), and validation (0.985) stages, and the RSE approaches zero (RSE = 0.033 for training, 0.028 for testing, and 0.03 for validation set), showing a strong prediction capability for the neural networks. Similarly, the other error metrics (MAE and RMSE) are also lower compared with the actual experimental values in the database and are almost consistent in each stage. Thus, the developed ANN model has reliable and suitable performance in the prediction of M_r_. However, it is hard to extract a proposed empirical formulation from the neural network algorithms due to their black box nature, restricting its wide-scale adoption [89,99].

#### 3.5.2. GEP Model

Like ANN, as presented in Table 5, the magnitude of R_GEP_ of the proposed model is greater than 0.8 but lower than that of the ANN predictive model for each subset of data. The R values for the training, testing, and validation stages are 0.86, 0.89, and 0.88, respectively, with the lower RSE metric equaling 0.37, 0.32, and 0.29, respectively. The RMSEs of both the predictive models are closer to each other in all three stages, showing that both models have almost an equal amount of higher error value. Moreover, the MAE statistic of GEP was higher as compared with ANN, giving the leading place to the ANN predictive model. However, the GEP algorithm along with an acceptable performance measure also gives a mathematical empirical model, which can be used to find the targeted vales independently.

### 3.6. Comparison of the Models

**Figure 8:**
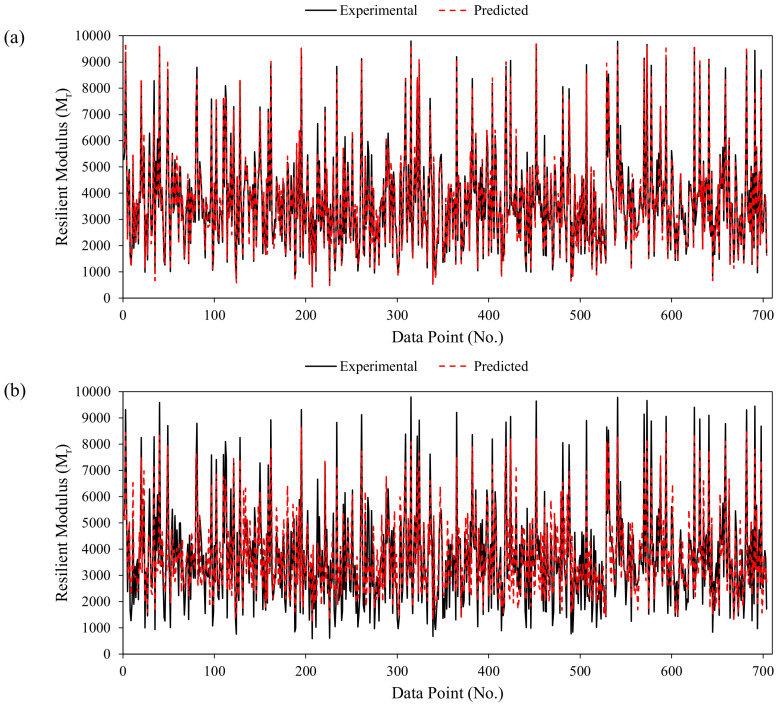
Comparison of the proposed models: (**a**) ANN; (**b**) GEP.

The predictive results of the ANN and GEP models for each single data point are graphically presented in Figure 8a,b, respectively. The experimental and predicted values by ANN are seen to largely diverge as compared with the GEP model. In each case (ANN and GEP), the predicted values clearly follow the actual experimental results and remain closer to each other. In accordance with the R value, the performances of the ANN model in the training, testing, and validation sets are 12.5%, 9.74%, and 10.65% better than the proposed GEP mathematical model. Although, the RMSE statistics of both models are almost similar in each stage. The comparative analysis of the ANN and GEP validates the fact that the ANN prediction is more accurate than the GEP model. The superior performance of the ANN is attributable to the complex computation capability of the ANN algorithmic structure while training the model [99,100]. However, the GEP is efficient in providing a mathematical equation with an acceptable performance measure. The proposed GEP equation can be used for future prediction of the M_r_ within the range of the input variables shown in Table 1. The GEP may serve as an appropriate and applicable modelling technique, and it may create a new domain for the reliable, effective, and accurate explicit formulation of several civil complex engineering problems; therefore, it can be utilized by any design practitioner or consultant without requiring familiarity with GEP.

### 3.7. Comparison of the Models

While comparing the developed ANN and GEP models with the existing literature, it was observed that a similar type of data were modelled using an ANN and an ELM optimized by PSO and a kernel-ELM (k-ELM). The training phases depicted R^2^ of 0.981, 0.693, and 0.64 for PSO-ELM, K-ELM, and PSO-ANN, respectively. The testing phase revealed an accuracy slightly smaller than that of the training phase. It is evident that the models developed earlier showed reliable comparable performance in relation to the current models; however, the GEP model presented in the current study expresses M_r_ in terms of input variables in the form of simple mathematical equation. Additionally, the optimal model obtained here was also utilized for performing both parametric and sensitivity analyses, which showed the contribution of each input parameter in yielding the M_r_ value. The GEP model presented here can be efficiently used for practical implications of the input variables while in the design and construction phases for different pavements. 

## 4. Concluding Remarks

For obtaining greater stiffness of subgrade materials under asphalt layers, aggregates are stabilized using calcium oxides and other cementitious materials. Resistance against wet–dry cycles (WDCs) is an important durability parameter for subgrade materials. This study investigates prediction models for estimating the resilient modulus based on the number of WDCs, the calcium oxide to SAF (silica, alumina, and ferric oxide compounds in the cementitious materials) ratio (CSAFR), the ratio of maximum dry density to the optimum moisture content (DMR), the confining pressure (σ_3_), and the deviator stress (σ_4_). The following conclusions can be drawn from this study.

- The Pearson’s linear correlation obtained for the experimental data showed that WDC showed a negative correlation, and CSAFR and DMR depicted a strong positive correlation with the resilient modulus (M_r_). The σ_3_ and σ_4_ showed slight positive correlations. The results from the parametric and sensitivity analyses also reflected similar interpretations of these variables. The results were corroborated by the previous literature. Thus, the results of the Pearson’s correlation, the sensitivity, and the parametric analyses and the literature are in good agreement with each other, rendering the developed models reliable for future use.
- The ANN model yielded the slopes of the regression line as 0.96, 0.99, and 0.94 for the training, validation, and testing data, respectively, in comparison with 0.72, 0.72, and 0.76, respectively, in the case of the GEP model. Values for R, MAE, and RMSE of 0.983, 245, and 60.52, respectively, were reported for ANN, whereas the GEP model manifested 0.86, 764 kPa, and 60.6 kPa, respectively, for the training data. The ANN model exceeded in accuracy in comparison with the GEP model.
- The sensitivity analysis revealed that DMR was the most influential parameter in contributing to M_r_ in both the models. Additionally, the CSAFR and WDC were reported as the next most important variables in the ANN modelling, whereas the WDC and CSAFR governed in the case of the GEP model. The σ_3_ and σ_4_ exhibited the least importance in estimating the M_r_ value. The parametric analysis of both the models showed that the M_r_ increased with DMR, σ_3_, and σ_4_. An increase in the number of the WDCs reduced the M_r_ value.

A variety of civil engineers and practitioners could utilize these easy-to-use mathematical expressions (attained from GEP modelling) during the design stage of a project or on site, preventing laborious and expensive laboratory testing for the determination of M_r_. The existing study is only valid for the given range of input and output parameters, and further studies need to be conducted considering an even wider range between the maximum and minimum values, and increasing the number of input parameters. In addition, the results could be optimized using the latest available optimization techniques, such as PSO, GWO, and SMA. 

## Figures and Tables

**Table 1:** Description of input and target parameters for model development.

	Variable	Description	Unit	Min	Max	Mean	Standard Deviation	Range
Inputs	WDC	Wet–dry cycle	-	0	30	12.795	11.158	30
	CSAFR	Calcium oxide to SAF ratio	-	0.113	0.51	0.255	0.183	0.397
	DMR	Ratio of maximum dry density to the optimum moisture content	kg·m^−3^	2.34	4.63	3.266	0.712	2.29
	σ_3_	Confining pressure	kPa	0	138	70.127	48.864	138
	σ_4_	Deviator stress	kPa	69	277	171.818	77.638	208
Target	M_r_	Resilient modulus	kPa	585	9803	3684.058	1860.495	9218

**Table 2:** Linear Pearson’s correlation indices for the inputs and the target variable considered in this study.

	WDC	CSAFR	DMR	σ_3_	σ_4_	M_r_
WDC	1	−0.05152	−0.01054	0.004294	0.016821	−0.29605
CSAFR	−0.05152	1	0.27031	0.013486	−0.01867	0.457157
DMR	−0.01054	0.27031	1	0.006829	−0.0216	0.714551
σ_3_	0.004294	0.013486	0.006829	1	−0.0019	0.076791
σ_4_	0.016821	−0.01867	−0.0216	−0.0019	1	0.137871
M_r_	−0.29605	0.457157	0.714551	0.076791	0.137871	1

**Table 3:** Setting parameters for the ANN model.

Parameter	Setting
Sampling	
Training records	492
Validation/testing	212
General	
Type	Input–output and curve fitting
Number of hidden neurons	10
Training Algorithm	Levenberg–Marquardt
Maximum Iterations	1000
Data division	Random

**Table 4:** Details of trials undertaken for selecting hyperparameters of GEP model.

Trial No.	Total Datasets	No. of Inputs	Fitness Function	No. of Chromosomes	No. of Genes	Head Size	Order of Variable Importance	Training Dataset		Validation Data	
								R	MAE	R	MAE
1	704	5	RMSE	30	3	8	32154	0.83	748	0.827	814
2					4		31452	0.854	783	0.89	743
3					5		31425	0.86	764	0.89	742
4				100	4	10	31245	0.85	790	0.877	782
5					5		32154	0.82	829	0.85	805
6			MAE				32154	0.8	806	0.82	800
7			RSE				31254	0.85	776	0.87	794

**Table 5:** Statistical evaluation of the developed models.

Model	Statistical Parameter	Training Set	Testing Set	Validation Set
ANN	MAE	245	255	227
	R	0.983	0.986	0.985
	RSE	0.033	0.028	0.03
	RMSE	60.52	62.03	61.42
GEP	MAE	764	742	743
	R	0.86	0.89	0.88
	RSE	0.37	0.32	0.29
	RMSE	60.6	62.31	60.81

## Data Availability

The data used in this research has been properly cited and reported in the main text.

## Abbreviations

AI	artificial intelligence
ANFIS	adaptive neuro-fuzzy inference system
ANN	artificial neural network
BBO	biogeography-based optimization
CSAFR	calcium oxide to (silica, alumina, and ferric oxide compounds) ratio
DMR	density to moisture content ratio
ELM	extreme learning machine
EO	equilibrium optimizer
ETs	expression trees
FTCs	freeze–thaw cycles
GA	genetic algorithm
GEP	gene expression programming
GP	gene programming
IIF	importance of input variables in percentage
MAE	mean absolute error
MEPDG	mechanistic empirical pavement design guidelines
PSO	particle swarm optimization
R	correlation coefficient
r	Pearson correlation coefficient
RF	random forest
RMSE	root mean squared error
RSE	root squared error
WDCs	wet–dry cycles
M_r_	resilient modulus
σ_3_	confining stress
σ_4_	deviator stress
